# Supplementary material for: Network Pharmacology and Experimental Validation to Investigate the Antidepressant Potential of Atractylodes lancea (Thunb.) DC
Source: Life (Basel). 2022 Nov 18;12(11):1925. doi: 10.3390/life12111925 (PMC9696776; doi:10.3390/life12111925)
Supplement: Supplementary file 1 [file life-12-01925-s001.zip › life-2025805-supplementary.pdf]

Table S1. Constituents of AL from TCMSP and literature.

Mol ID	Molecule Name	MW	OB (%)	Caco-2	BBB	DL	Source
MOL013068	Oroxindin	459.41	7.07	-1.68	-2.15	0.77	TCMSP
MOL000184	Stigmastenone	412.77	39.25	1.42	1.22	0.76	TCMSP
MOL000186	Stigmasterol 3-O-β-D-glucopyranoside_qt	412.77	43.83	1.31	0.9	0.76	TCMSP
MOL000094	Daucosterol_qt	414.79	36.91	1.3	0.87	0.76	TCMSP
MOL000092	Daucosterin_qt	414.79	36.91	1.42	1.15	0.76	TCMSP
MOL000449	Stigmasterol	412.77	43.83	1.44	1	0.76	[1]
MOL000088	β-sitosterol 3-O-glucoside_qt	414.79	36.91	1.3	0.91	0.75	TCMSP
MOL000085	β-daucosterol_qt	414.79	36.91	1.3	0.88	0.75	TCMSP
MOL000095	Δ-7-stigmastenol	416.81	25.32	1.31	0.98	0.75	TCMSP
MOL000086	(24S)-5β-Stigmastan-3β-ol	416.81	25.32	1.41	1.18	0.75	TCMSP
MOL000358	β-sitosterol	414.79	36.91	1.32	0.99	0.75	[1]
MOL000185	Stigmasterol 3-O-β-D-glucopyranoside	574.93	21.32	-0.22	-0.93	0.63	TCMSP
MOL000084	β-daucosterol	576.95	20.63	-0.19	-0.88	0.63	TCMSP
MOL000093	Daucosterol	576.95	20.63	-0.26	-0.97	0.63	TCMSP
MOL000091	Daucosterin	576.95	20.63	-0.28	-1	0.63	TCMSP
MOL000087	β-sitosterol 3-O-glucoside	576.95	20.63	-0.45	-1.18	0.62	TCMSP
MOL000192	2-(1,4a-dimethyl-2,3-dihydroxydecahydronaphthalen-7-yl) isopropyl glucoside	418.59	5.96	-1.12	-1.86	0.47	TCMSP
MOL000190	3,5-dimethoxy-4-glucosyloxyphenylallylalcohol	372.41	29	-1.04	-1.59	0.32	TCMSP
MOL000173	Wogonin	284.28	30.68	0.79	0.04	0.23	TCMSP
MOL000182	Atractyloyne	314.46	5.2	0.73	-0.98	0.23	TCMSP
MOL000188	3β-acetoxyatractylone	274.39	40.57	1.22	1.04	0.22	TCMSP
MOL000179	2-Hydroxyisoxypropyl-3-hydroxy-7-isopentene-2,3-dihydrobenzofuran-5-carboxylic	306.39	45.2	-0.34	-0.91	0.2	TCMSP
MOL000178	Atractylenolide III	248.35	31.66	0.75	0.64	0.17	TCMSP
MOL000187	Butenolide B	234.32	61	0.65	0.45	0.15	TCMSP
MOL000043	Atractylenolide I	230.33	37.37	1.3	1.29	0.15	TCMSP
MOL000167	3β-hydroxyatractylone	232.35	21.17	1.18	0.97	0.15	TCMSP
MOL000044	Atractylenolide II	232.35	47.5	1.3	1.37	0.15	TCMSP

Table S1. (continued)

Mol ID	Molecule Name	MW	OB (%)	Caco-2	BBB	DL	Source
MOL000164	Atractylone	216.35	33.91	1.74	1.83	0.13	TCMSP
MOL000189	Acetyl atractylodinol	240.27	25.47	1.42	0.69	0.13	TCMSP
MOL000194	Patchoulene	204.39	51.71	1.8	2.21	0.11	TCMSP
MOL000175	Cyperene	204.39	51.1	1.81	2.13	0.11	TCMSP
MOL000060	Selina-4(14),7(11)-dien-8-one	218.37	32.31	1.42	1.57	0.1	TCMSP
MOL000032	β-Eudesmol	222.41	26.09	1.32	1.38	0.1	TCMSP
MOL000193	(Z)-caryophyllene	204.39	30.29	1.82	2.15	0.09	TCMSP
MOL000165	2-[(2S,5S,6S)-6,10-dimethylspiro[4.5]dec-9-en-2-yl]propan-2-ol	222.41	37.62	1.44	1.53	0.09	TCMSP
MOL000171	Guaiol	222.41	38.77	1.36	1.47	0.09	TCMSP
MOL000058	Hinesol	222.41	38.59	1.34	1.42	0.09	[1-3]
MOL000162	β-Chamigrene	204.39	31.99	1.82	2.07	0.08	TCMSP
MOL000034	2-[(1R,3S,4S)-3-isopropenyl-4-methyl-4-vinylcyclohexyl]propan-2-ol	222.41	19.03	1.37	1.46	0.07	TCMSP
MOL000169	α-Guaiene	204.39	25.93	1.81	2.09	0.07	TCMSP
MOL000170	Guaiene	204.39	28.21	1.83	2.03	0.07	TCMSP
MOL000174	(2E,8E)-9-(2-furyl)nona-2,8-dien-4,6-diyn-1-ol	198.23	47.6	1.33	0.14	0.07	TCMSP
MOL000166	ZINC01609418	222.41	21.62	1.41	1.37	0.07	TCMSP
MOL012364	Elemol	222.41	31.91	1.28	1.32	0.07	[1]
MOL000191	3,5-dimethoxy-4-glucosyloxyphenylallylalcohol_qt	210.25	72.16	0.61	0.2	0.06	TCMSP
MOL000024	α-humulene	204.39	22.98	1.88	2.08	0.06	TCMSP
MOL000027	α-Curcumene	202.37	4.68	1.93	1.99	0.06	[3]
MOL000163	Atractylodin	182.23	44.49	2	1.03	0.05	TCMSP
MOL000168	()-2-Carene	136.26	46.69	1.89	2.27	0.04	TCMSP
MOL000114	Vanillic acid	168.16	35.47	0.43	0.09	0.04	TCMSP
MOL000172	Furol	96.09	34.35	1.08	1.51	0.01	TCMSP

Table S2. Targets of AL compounds.

Gene	Compound
ACHE	MOL000188
ADH1C	MOL000449
ADRA1A	MOL000188, MOL000449, MOL000358
ADRA1B	MOL000449, MOL000194, MOL000358
ADRA2A	MOL000449
ADRB1	MOL000449
ADRB2	MOL000188, MOL000173, MOL000449, MOL000358
AHSA1	MOL000173
AKR1B1	MOL000449
AR	MOL000173, MOL000188,
BAX	MOL000173, MOL000358
BBC3	MOL000173
BCL2	MOL000173, MOL000358
CALM2	MOL000173
CASP3	MOL000173, MOL000358
CASP8	MOL000358
CASP9	MOL000173, MOL000358
CCL2	MOL000173
CCND1	MOL000173
CDK2	MOL000173
CDKN1B	MOL000173
CHEK1	MOL000173
CHRM1	MOL000188, MOL000060, MOL000449, MOL000194, MOL000358
CHRM2	MOL000188, MOL000060, MOL000449, MOL000194, MOL000358
CHRM3	MOL000188, MOL000060, MOL000449, MOL000194, MOL000358, MOL000175
CHRM4	MOL000358
CHRNA2	MOL000358, MOL000194
CHRNA7	MOL000060, MOL000032, MOL000449, MOL000178, MOL000043, MOL000044, MOL000358
CTRB1	MOL000449
CYP101	MOL000358
DPP4	MOL000188, MOL000173
DRD1	MOL000188, MOL000358

Table S2. (continued)

Gene	Compound
EIF6	MOL000173
ESR1	MOL000173
F2	MOL000188
FN1	MOL000173
GABRA1	MOL000175, MOL000060, MOL000188, MOL000449, MOL000358, MOL000173, MOL000178, MOL000043, MOL000044, MOL000194, MOL000032
GABRA2	MOL000060, MOL000043, MOL000044, MOL000358
GABRA3	MOL000060, MOL000044, MOL000449, MOL000358
GABRA5	MOL000358
GABRA6	MOL000060, MOL000178
GRM2	MOL000178, MOL000044
GSK3B	MOL000173
HSP90	MOL000358, MOL000173
HTR2A	MOL000188, MOL000449, MOL000358
IGHG1	MOL000449
IL1B	MOL000043
IL6	MOL000173, MOL000043
IL8	MOL000173
JUN	MOL000358, MOL000173
KCNH2	MOL000358
LTA4H	MOL000449
MAOA	MOL000449
MAOB	MOL000449
MAP2	MOL000358
MAPK14	MOL000173
MCL1	MOL000173
MMP1	MOL000173
NCOA1	MOL000449
NCOA2	MOL000175, MOL000449, MOL000358, MOL000194
NOS2	MOL000173
NOS3	MOL000188
NR3C2	MOL000449
OPRM1	MOL000188, MOL000358

Table S2. (continued)

Gene	Compound
TEP1	MOL000173
TGFB1	MOL000358
TNF	MOL000043, MOL000173
TP53	MOL000173
VEGFA	MOL000043
VEGFR2	MOL000173
PDE3A	MOL000188, MOL000358, MOL000173
PGF	MOL000043
PGR	MOL000449, MOL000184, MOL000085, MOL000095, MOL000086
PIK3CG	MOL000358, MOL000173
PLAU	MOL000449
PON1	MOL000358
PPARG	MOL000173
PRKACA	MOL000188, MOL000449, MOL000173
PRKCA	MOL000358
PRKCD	MOL000173
PRSS1	MOL000173
PTGER3	MOL000173
PTGS1	MOL000449, MOL000358, MOL000173
PTGS2	MOL000175, MOL000060, MOL000188, MOL000449, MOL000358, MOL000173
RELA	MOL000173
RXRA	MOL000188, MOL000449, MOL000173
SCN5A	MOL000188, MOL000449, MOL000358, MOL000173
SLC6A2	MOL000060, MOL000188, MOL000449
SLC6A3	MOL000188, MOL000449
SLC6A4	MOL000188, MOL000358

Table S3. 260 genes associated with depression acquired from DisGeNET

(Disease ID: C0011570, score ≥ 0.3)

Depression-related targets						
A2M	*CDKN2A*	*DPYSL2*	*GRIN2A*	*IL1A*	*NTRK2*	*SFRP1*
AANAT	*CHAT*	*DRD1*	*GRIN2B*	*IL1B*	*NTS*	*SGCE*
ABCB1	*CHRM2*	*DRD2*	*GRK2*	*IL6*	*OAS2*	*SLC18A1*
ADCY5	*CHRNA2*	*DRD3*	*GRM1*	*IL6R*	*OPRK1*	*SLC18A2*
ADCY7	*CHRNA4*	*DRD4*	*GRM7*	*IMPA2*	*OPRM1*	*SLC1A1*
ADCY8	*CHRNA6*	*DTNBP1*	*GRN*	*KCNJ6*	*OR7D4*	*SLC1A2*
ADCYAP1	*CHRNB3*	*DUSP1*	*GRPR*	*KCNK2*	*OXT*	*SLC29A3*
ADCYAP1R1	*CLOCK*	*DUSP4*	*GSK3B*	*LDHA*	*OXTR*	*SLC6A1*
ADRA2A	*CMKLR1*	*DUSP6*	*GSTM1*	*LEP*	*P2RX7*	*SLC6A2*
ADRB1	*CNR1*	*EGR3*	*GSTT1*	*LGI1*	*PCLO*	*SLC6A3*
AGO1	*CNR2*	*ERBB3*	*GTS*	*LIF*	*PDE1B*	*SLC6A4*
AGT	*CNTF*	*ESR1*	*GYPE*	*LTA4H*	*PDE4A*	*SLCO1C1*
AKT1	*COMT*	*FEV*	*HCN1*	*M6PR*	*PDE4B*	*SNAP25*
ALK	*COX2*	*FGF17*	*HCRT*	*MAOA*	*PDE4D*	*SNCA*
ANKK1	*CPLX1*	*FGF20*	*HCRTR1*	*MAOB*	*PDYN*	*SOD1*
APP	*CPLX2*	*FGFR1*	*HDAC2*	*MAPK3*	*PENK*	*SOD2*
APRT	*CREB1*	*FGFR2*	*HDAC4*	*MAPK8*	*PER2*	*SRD5A1*
AQP4	*CRH*	*FKBP5*	*HDAC5*	*MC1R*	*PER3*	*STMN1*
AR	*CRHBP*	*FOLH1*	*HDAC6*	*MC4R*	*PEX5L*	*SYN1*
ARHGEF10	*CRHR1*	*FOS*	*HDAC9*	*MCHR1*	*PFKFB3*	*TAC1*
ARNTL	*CRHR2*	*FTO*	*HIF1A*	*MED12*	*PMCH*	*TACR1*
ARRB2	*CRY1*	*GABRA3*	*HOMER1*	*MTHFR*	*PNOC*	*TBX19*
ARTN	*CRY2*	*GABRA6*	*HP*	*MTR*	*POMC*	*TH*
ASMT	*CSMD2*	*GABRB3*	*HSD11B1*	*NCAM1*	*PPP1R1B*	*TIMELESS*
ATF3	*CXCL8*	*GAD1*	*HTR1A*	*NEFM*	*PPP3CC*	*TLE1*
ATF4	*CYP2C19*	*GAL*	*HTR1B*	*NGF*	*PRKCI*	*TNF*
ATP1A3	*CYP2D6*	*GAP43*	*HTR2A*	*NGFR*	*PSEN1*	*TNFRSF1A*
ATP2A2	*DAOA*	*GFAP*	*HTR2C*	*NOS2*	*PTGS2*	*TNFRSF1B*
ATXN3	*DAOA-AS1*	*GH1*	*HTR3A*	*NOS3*	*PYY*	*TPH1*
BAG1	*DBH*	*GLO1*	*HTR3B*	*NPAS2*	*RAC1*	*TPH2*
BDNF	*DEAF1*	*GLUL*	*HTR4*	*NPS*	*RELN*	*TRH*
BICC1	*DGCR8*	*GNB3*	*HTR7*	*NPSR1*	*REN*	*TTR*
BRCA1	*DGKB*	*GPX1*	*HTT*	*NPY*	*RNF123*	*UCN*
CACNA1C	*DISC1*	*GRIA1*	*IDO1*	*NR3C1*	*RORA*	*VEGFA*
CALM2	*DKK4*	*GRIA3*	*IFNG*	*NR3C2*	*S100A10*	*VGF*
CAMK2A	*DLG4*	*GRID1*	*IGF1*	*NRG1*	*S100B*	*WFS1*
CARTPT	*DPP4*	*GRIK3*	*IL18*	*NRXN1*	*SERPINA6*	*WWC1*
CDH13						

Table S4. Gene Ontology (GO) Biological Process analysis on the potential targets of AL compounds.

GO Biological Process	Related genes	Combined score
regulation of peptide hormone secretion (GO:0090276)	*DPP4, IL6, NOS2, IL1B, TNF, ADRA2A*	1678.71
phospholipase C-activating G protein-coupled receptor signaling pathway (GO:0007200)	*CHRM2, HTR2A, DRD1, OPRM1, ESR1, ADRA2A*	1481.4
positive regulation of acute inflammatory response (GO:0002675)	*IL6, IL1B, PTGS2, TNF*	5837.22
regulation of insulin secretion (GO:0050796)	*DPP4, IL6, NOS2, IL1B, TNF, ADRA2A*	1048.31
regulation of neuroinflammatory response (GO:0150077)	*IL6, IL1B, TNF, PTGS2*	4049.2
cellular response to cytokine stimulus (GO:0071345)	*GSK3B, IL6, NOS2, MAOA, IL1B, OPRM1, PTGS2, TNF, VEGFA*	356.64
positive regulation of gene expression (GO:0010628)	*GSK3B, AR, IL6, NOS3, IL1B, DRD1, TNF, ADRA2A, VEGFA*	356.64
regulation of protein secretion (GO:0050708)	*DPP4, IL6, NOS2, IL1B, TNF, ADRA2A*	811.96
regulation of fever generation (GO:0031620)	*IL1B, PTGS2, TNF*	20996.79
positive regulation of glial cell proliferation (GO:0060252)	*IL6, IL1B, TNF*	20996.79

Table S5. Gene Ontology (GO) Molecular Function analysis on the potential targets of AL compounds.

GO Molecular Function	Related genes	Combined score
sodium:chloride symporter activity (GO:0015378)	*SLC6A2, SLC6A3, SLC6A4*	6149.17
monoamine transmembrane transporter activity (GO:0008504)	*SLC6A2, SLC6A3, SLC6A4*	6149.17
transmitter-gated ion channel activity involved in regulation of postsynaptic membrane potential (GO:1904315)	*CHRNA2, GABRA6, GABRA3*	1023.01
RNA polymerase II general transcription initiation factor binding (GO:0001091)	*AR, ESR1*	5570.3
arginine binding (GO:0034618)	*NOS2, NOS3*	2521.84
receptor ligand activity (GO:0048018)	*DPP4, IL6, TNF, IL1B, VEGFA*	137.49
benzodiazepine receptor activity (GO:0008503)	*GABRA6, GABRA3*	1800.45
cytokine activity (GO:0005125)	*IL6, IL1B, TNF, VEGFA*	181.06
extracellular ligand-gated ion channel activity (GO:0005230)	*GABRA6, GABRA3*	1566.22
FMN binding (GO:0010181)	*NOS2, NOS3*	1566.22

Table S6. Gene Ontology (GO) Cellular Component analysis on the potential targets of AL compounds.

GO Cellular Component	Related genes	Combined score
neuron projection (GO:0043005)	*CHRM2, GSK3B, CHRNA2, GABRA3, HTR2A, OPRM1, PTGS2, SLC6A2, SLC6A3, SLC6A4*	531.75
integral component of plasma membrane (GO:0005887)	*CHRM2, CHRNA2, GABRA6, GABRA3, ADRB1, HTR2A, OPRM1, SLC6A2, TNF, SLC6A3, ADRA2A, SLC6A4, IL6, DRD1*	260.27
dendrite (GO:0030425)	*CHRM2, GSK3B, GABRA6, GABRA3, HTR2A, OPRM1*	270.7
GABA-A receptor complex (GO:1902711)	*GABRA6, GABRA3*	727.05
dendrite membrane (GO:0032590)	*GABRA6, GABRA3*	408.9
organelle outer membrane (GO:0031968)	*MAOA, MAOB, PTGS2*	118.14
membrane raft (GO:0045121)	*TNF, SLC6A3, SLC6A4*	96.63
axon (GO:0030424)	*GSK3B, OPRM1, SLC6A3*	69.22
caveola (GO:0005901)	*NOS3, SLC6A3*	151.87
vesicle (GO:0031982)	*NOS2, NOS3, CALM2*	59.26

Table S7. Kyoto Encyclopedia Genes and Genomes (KEGG) pathway analysis on the potential targets of AL compounds.

**KEGG pathway**	**Related genes**	**Combined score**
Neuroactive ligand-receptor interaction	*CHRM2, CHRNA2, GABRA6, GABRA3, ADRB1, HTR2A, DRD1, OPRM1, ADRA2A*	596.5
Calcium signaling pathway	*CHRM2, NOS2, ADRB1, HTR2A, DRD1, CALM2, VEGFA*	706.46
Human cytomegalovirus infection	*GSK3B, IL6, IL1B, PTGS2, TNF, CALM2, VEGFA*	535.28
Dopaminergic synapse	*GSK3B, MAOA, MAOB, DRD1, CALM2, SLC6A3*	752.54
Amphetamine addiction	*MAOA, MAOB, DRD1, CALM2, SLC6A3*	1153.67
Pertussis	*IL6, IL1B, NOS2, TNF, CALM2*	1009.69
IL-17 signaling pathway	*GSK3B, IL6, IL1B, PTGS2, TNF*	752.7
Pathways of neurodegeneration	*GSK3B, IL6, NOS2, IL1B, PTGS2, TNF, CALM2, SLC6A3*	258.09
AGE-RAGE signaling pathway in diabetic complications	*IL6, NOS3, IL1B, TNF, VEGFA*	690.84
C-type lectin receptor signaling pathway	*IL6, IL1B, PTGS2, TNF, CALM2*	654.23
Lipid and atherosclerosis	*GSK3B, IL6, NOS3, IL1B, TNF, CALM2*	377.17
Pathways in cancer	*GSK3B, AR, IL6, NOS2, PTGS2, ESR1, CALM2, VEGFA*	217.19
Serotonergic synapse	*MAOA, MAOB, HTR2, PTGS2, SLC6A4*	582.88
Alzheimer disease	*GSK3B, IL6, NOS2, IL1B, PTGS2, TNF, CALM2*	259.22
Cocaine addiction	*MAOA, MAOB, DRD1, SLC6A3*	1054.52
Arginine and proline metabolism	*MAOA, MAOB. NOS2, NOS3*	1025.6
Fluid shear stress and atherosclerosis	*NOS3, IL1B, TNF, CALM2, VEGFA*	436.1
Leishmaniasis	*NOS2, IL1B, PTGS2, TNF*	566.11
Tuberculosis	*IL6, NOS2, IL1B, TNF, CALM2*	302.01
Alcoholism	*MAOA, MAOB, DRD1, CALM2, SLC6A3*	288.11

Table S8. Docking scores of compounds in AL and potential targets.

	Docking score (kcal/mol)								
**Compound**	**CHMR2**	**ESR1**	**GABRA3**	**HTR2A**	**IL6**	**NOS3**	**PTGS2**	**SCL6A4**	**TNF**
3β-acetoxyatractylone	−7.7	-	-	−6.8	-	-7.8	−8.4	−7.2	-
Atractylenolide I	-	-	-	-	−6.6	-	-	-	−7.7
Atractylenolide II	-	-	−7.3	-	-	-	-	-	-
Cyperene	-	-	-	-	-	-	−7.0	-	-
Patchoulene	−7.2	-	-	-	-	-	-	-	-
Selina-4(14),7(11)-dien-8-one	−7.1	-	−6.7	-	-	-	−7.3	-	-
Stigmasterol	−8.6	-	−8.0	−9.0	-	-	−9.9	-	-
Wogonin	-	−6.7	-	-	−6.3	-	−8.0	-	−6.4
β-sitosterol	−8.7	-	−7.7	−8.6	-	-	−9.4	−6.8	-

References

1. Jun, X.; Fu, P.; Lei, Y.; Cheng, P. Pharmacological effects of medicinal components of Atractylodes lancea (Thunb.) DC. *Chin. Med.* **2018,** *13*, 59.
2. Koonrungsesomboon, N.; Na-Bangchang, K.; Karbwang, J. Therapeutic potential and pharmacological activities of Atractylodes lancea (Thunb.) DC. *Asian Pac. J. Trop. Med.* **2014,** *7*, 421–428.
3. Cheng, Y.; Mai, J. Y.; Hou, T. L.; Ping, J.; Chen, J. J. Antiviral activities of atractylon from Atractylodis Rhizoma. *Mol. Med. Rep.* **2016,** *14*, 3704–3710.
